# Embedding gene trees into phylogenetic networks by conflict resolution algorithms

**DOI:** 10.1186/s13015-022-00218-8

**Published:** 2022-05-19

**Authors:** Marcin Wawerka, Dawid Dąbkowski, Natalia Rutecka, Agnieszka Mykowiecka, Paweł Górecki

**Affiliations:** University of Warsaw, Faculty of Mathematics, Informatics and Mechanics, Banacha 2, 02-097 Warsaw, Poland

**Keywords:** Phylogenetic network, Tree-child network, Gene tree, Species tree, Deep coalescence, Reticulation, Optimal displayed tree

## Abstract

**Background:**

Phylogenetic networks are mathematical models of evolutionary processes involving reticulate events such as hybridization, recombination, or horizontal gene transfer. One of the crucial notions in phylogenetic network modelling is displayed tree, which is obtained from a network by removing a set of reticulation edges. Displayed trees may represent an evolutionary history of a gene family if the evolution is shaped by reticulation events.

**Results:**

We address the problem of inferring an optimal tree displayed by a network, given a gene tree *G* and a tree-child network *N*, under the deep coalescence and duplication costs. We propose an *O*(*mn*)-time dynamic programming algorithm (DP) to compute a lower bound of the optimal displayed tree cost, where *m* and *n* are the sizes of *G* and *N*, respectively. In addition, our algorithm can verify whether the solution is exact. Moreover, it provides a set of reticulation edges corresponding to the obtained cost. If the cost is exact, the set induces an optimal displayed tree. Otherwise, the set contains pairs of conflicting edges, i.e., edges sharing a reticulation node. Next, we show a conflict resolution algorithm that requires $2^{r+1}-1$ invocations of DP in the worst case, where *r* is the number of reticulations. We propose a similar $O(2^kmn)$-time algorithm for level-*k* tree-child networks and a branch and bound solution to compute lower and upper bounds of optimal costs. We also extend the algorithms to a broader class of phylogenetic networks. Based on simulated data, the average runtime is $\Theta (2^{{0.543}k}mn)$ under the deep-coalescence cost and $\Theta (2^{{0.355}k}mn)$ under the duplication cost.

**Conclusions:**

Despite exponential complexity in the worst case, our algorithms perform significantly well on empirical and simulated datasets, due to the strategy of resolving internal dissimilarities between gene trees and networks. Therefore, the algorithms are efficient alternatives to enumeration strategies commonly proposed in the literature and enable analyses of complex networks with dozens of reticulations.

## Background

Evolutionary networks are mathematical models of evolutionary processes with reticulate events such as hybridization, recombination, or horizontal gene transfer [1, 2]. Hybridization is a common phenomenon in plants and is often used in agriculture to create new breeds [3]. Recombination and reassortment are two shuffling processes in which variants of genetic material are created from pairs of highly similar DNA sequences. For example, many viruses have segmented genomes, including influenza viruses and rotaviruses [4], while horizontal gene transfer is common in bacteria [5]. In the last decades, mathematical and computational properties of phylogenetic networks have been intensively studied (see books [2, 6]). One of the most classic notions is a *tree displayed by a network*, obtained from a network by removing a set of *reticulation edges*. Displayed trees may represent an evolutionary history of a gene family [2], if the evolution of genes and their species is shaped by reticulation events. Alternative approaches include embedding a gene tree into a displayed tree [7–9] or using a parental species tree as a generalization of a displayed tree [9–11].

The pioneering work by Maddison [12] introduced the deep coalescence (DC) cost, which measures the *extra* gene lineages of a gene tree when embedded into a species tree. When a gene is embedded into its species tree, each edge of the species contains several mapped gene lineages. For example, when both trees have the same topology, there are no extra gene lineages in the perfect situation. DC and general coalescent-based methods are popular in classical problems of computational biology, e.g., estimation of species trees [13–15], tree reconciliation [15–19], or gene tree error correction [20].

Goodman et al. [21] introduced a duplication model more than 40 years ago to explain potential discordance between the gene tree and the species tree originating from complex histories of gene duplication and loss events. This approach is based on embedding the gene tree into the species tree using a *mapping* [22] that relates every gene in the gene tree to its *host species* that is the most recent species that could have contained the gene. Consequently, the mapping relates every leaf-gene of the gene tree to the species from which it has been sampled. Based on this mapping, evolutionary events such as gene duplications are identified. A node in a gene tree denotes a *gene duplication* when it has a child with the same host species. While many embeddings are possible [23], the classic mapping describes the most parsimonious embedding in terms of the number of gene duplication and loss events [23, 24]. The gene duplication model has many theoretical and practical applications [15, 18, 25–27].

There are two main general approaches to embed a gene tree into a network using the parsimony principle: (1) choosing the tree displayed by the network with the lowest cost, i.e. solving the optimal displayed tree (ODT) problem, in which a reticulation node can be reached only from one fixed parent, or (2) *a direct tree-network* embedding, without the above constraint. These approaches are present in relevant articles concerning inferences of networks under Robinson-Foulds (RF) embedding cost [8], the duplication-loss cost [9], and the deep coalescence cost [7]. The latter includes the general parsimony framework using the concept of parental species trees [10]. Alternative studies are based on minimizing deep coalescence criterion [25] or on probabilistic models on coalescent histories [28]. Model-based approaches are usually computationally demanding since they often require enumeration of all possible coalescence histories [28, 29]. Finally, perhaps one of the most prominent applications of the above methods is the problem of network inference (e.g. [2, 8–10, 29, 30]).

From the theoretical point of view, ODT under deep coalescence (or duplication cost) corresponds to NP-hard problems: (1) best switching (i.e., choosing the set of reticulation edges) for the duplication-loss model [9], and (2) the computation of RF-embedding cost [8]. In [9], the problem is solved in $O(|N|+p2^k|G|)$ time, where *G* is a gene tree and *p* is the number of biconnected components in a level-*k* network *N*. [8] proposed an $O(2^r|N|)$-time optimized algorithm to compute RF-embedding cost, where *r* is the number of reticulations in *N*. Another relevant contribution is from [7] with an $O(4^k|G||N|^2)$-time tree vs. level-*k* network reconciliation algorithm under DC events. However, the latter cannot be directly compared to ours since we solve a different problem. In all of the above contributions, the complexity related to $2^r$ (or $2^k$) is reached due to exhaustive enumeration strategies. In this article, we show how to avoid such strategies by proposing an efficient in practice method to infer optimal displayed trees despite the theoretical intractability of ODT in general.

*Our contribution:* We address the problem of inference of an optimal tree displayed by a tree-child network (ODT), given a gene tree *G* and a tree-child network *N* under the deep coalescence (DC) and duplication (D) costs. We propose a novel approach in which we define scenarios for embedding *G* into *N* using sets of reticulation edges from *N*, with a property that the score of a scenario approximates the displayed tree cost. In particular, we prove that the score of a scenario is a lower bound of the cost of the optimal displayed tree. In a specific case, when a scenario induces a non-conflicting set of reticulation edges, we provide the correspondence between a score of this scenario and a cost of a displayed tree. Next, we propose an *O*(|*G*||*N*|) time dynamic programming (DP) algorithm to compute an optimal scenario. We show that an optimal scenario with no conflicts corresponds to a solution of ODT. Based on DP, we design a recursive algorithm to ODT by resolving conflicts in sets of reticulation edges. This algorithm has exponential time complexity $O(2^r|G||N|)$, where *r* is the number of reticulation nodes in *N*. We propose a similar $O(2^k|G||N|)$-time algorithm for level-*k* tree-child networks. We also show how the algorithms can be extended to a broader class of phylogenetic networks defined by the property: *each node has at most one reticulation child*. Finally, we show experimental studies on random, simulated, and empirical datasets. We show that our algorithm has significantly improved runtime on simulated datasets by reducing the exponent from *r* to nearly half of *r* on average.

## Methods

In this section, we present the main theoretical and algorithmic methods on the inference of an optimal tree displayed by a network problem (ODT). We mainly focus on the details related to the variant of the problem under the deep coalescence cost (ODT-DC). At the end of the section, we show how the theory can be adopted to solve the problem under the duplication cost (ODT-DUP).

### Trees and networks

A *network* on a set of species *X* is a directed acyclic graph $N=(V(N),E(N))$ with a single root such that: (1) its leaves, i.e., nodes of indegree 1 and outdegree 0, are labeled by the species from *X*, and (2) there is a directed path from the root to any other vertex. A network is *binary* if its leaves, root, and the remaining nodes have degrees 1, 2 and 3, respectively. A node is called a *reticulation* if it has indegree two and outdegree one, and a *tree node* if it has indegree at most one and outdegree two. A network is *semi-binary*, if additionally, it may contain *semi-binary nodes* of indegree at most one and outdegree one, which includes the root having exactly one child. We can *contract* a semi-binary node *v* of indegree one as follows: (1) remove *v*, (2) remove both edges incident with *v*, and (3) insert a new directed edge connecting the unique parent of *v* with the only child of *v*. Similarly, if *v* has indegree zero we remove *v*, and the child of *v* becomes a new root. If a directed graph $G'$ is obtained from a graph *G* by a sequence of contract operations, then *G* is called a *subdivision* of $G'$.

If $\langle v,w \rangle \in E(N)$, then *v* is a parent of *w* and *w* is a child of *v*, denoted $w.\mathsf {parent}=v$ if *w* is a non-root tree node or a leaf. We write $v.{\mathsf {sibling}}=w$ if $v \ne w$ have the same parent. We write $v \succeq w$ if there is a directed path from *v* to *w*, and $v \succ w$ if $v \succeq w$ and $v \ne w$. The set of all leaves in a network is denoted *L*(*N*), by $R(N) \subset V(N)$ we denote the set of reticulation nodes in *N*, by $T(N) \subset V(N)$ we denote the set of all tree nodes in *N*, and by $E_R(N) \subset E(N)$ we denote the set of all reticulation edges in *N*, that is, edges $\langle v,r \rangle \in E(N)$ with $r \in R(N)$. We say that a reticulation edge *e* is a *sibling* of a reticulation edge $e'$ if they share the same bottom reticulation node. By $\deg _N(v)$ we denote the outdegree of *v* in *N*.

A *phylogenetic network* is a binary network on *X* in which the leaves are labeled one-to-one with the species from *X*^1^. A *species tree* is a phylogenetic network without reticulation nodes. A *gene tree*, or in short *a tree*, is a binary network without reticulation nodes. Note that the leaf labeling in a gene tree does not have to be one-to-one. Such labelled trees are called multi-labelled trees or MUL-trees [31]. A phylogenetic network is *tree-child* network, if each non-leaf node has a child that is either a tree node or a leaf [32–35].

### Deep coalescence cost: embedding a tree into a (displayed) tree

Given a gene tree *G* and a species tree *S* on *X*, the *lca-mapping* $\mathsf {M}:V(G) \rightarrow V(S)$ is defined as follows: (1) if *g* is a leaf labeled $x \in X$ then $\mathsf {M}(g)$ is the unique leaf labeled *x* in *S*, and (2) if *g* has two children $g'$ and $g''$, then $\mathsf {M}(g)$ is the lowest common ancestor of $\mathsf {M}(g')$ and $\mathsf {M}(g'')$ in *S*. Embedding *G* into *S* is performed by mapping each edge $\langle v,w \rangle \in E(G)$ to a path connecting $\mathsf {M}(v)$ and $\mathsf {M}(w)$ in *S*. We say that the gene edge *visits* edges from that path. Let ||*v*, *w*|| denote the number of edges on the path connecting *v* and *w*. Then, the visited edges contribute to the deep coalescence cost, denoted $\mathsf {DC}(G,S)$, as follows:

1 $$\begin{aligned} \mathsf {DC}(G,S) = \sum _{\langle v,w \rangle \in E(G)} (||\mathsf {M}(v),\mathsf {M}(w)||-1). \end{aligned}$$

Given a phylogenetic network *N* on *X*, we say that a species tree *T* on *X* is *displayed* by *N*, if *N* contains a subgraph $T'$ that is a subdivision of *T* [36].

We now define the *Optimal Displayed Tree under Deep Coalescence* problem (ODT-DC) in the parsimony framework:

#### Problem 1

(ODT-DC) Given a tree *G* and a phylogenetic network *N*. Find an optimal tree $S^*$ displayed by *N* that minimizes $\mathsf {DC}(G,S)$ in the set of all trees *S* displayed by *N*.

**Fig. 1:**
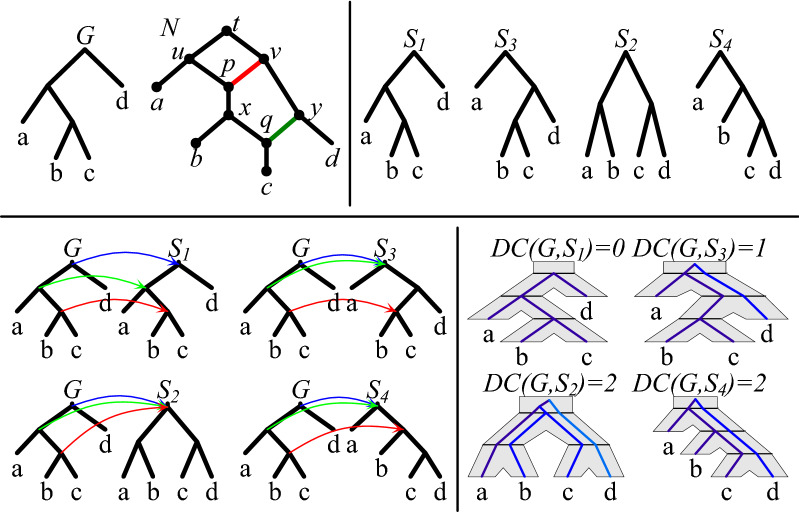
Top left: a gene tree *G* and a phylogenetic network *N* with two reticulations. Top right: four trees displayed by *N*. Bottom: lca-mappings between *G* and $S_i$’s, visualization of embeddings of *G* into $S_i$’s. Here, $S_1$ is the optimal tree displayed by *N* with the $\mathsf {DC}$ cost of 0.

The *cost* of an optimal displayed tree, we denote $\mathsf {DC}(G,N)$. While the complexity of ODT-DC remains unknown for the class of tree-child networks, we claim that the problem is NP-hard in a general class of networks. The proof is similar to the NP-hardness proof of the best switching problem from [9]. See also [8] for the related problem of RF-embedding. Figure 1 depicts an example of DC costs.

### Scenarios between gene trees and phylogenetic networks

In the previous Section, we showed how a gene tree is embedded into a species tree. Here, we propose to embed a gene tree into a phylogenetic network using a more general approach than embedding through a displayed tree. We start with the notion of *unfolded network* (see also [37, 38]), then we define *scenarios* between gene trees and unfolded networks.

For a phylogenetic network *N* on *X* with *k* reticulations, the *unfolded network* $\hat{N}$ is the tree $N_k$ obtained from *N* by a sequence of *k* unfolding operations defined on pairs $(N_i,\sigma _i)$, such that $N_i$ is a semi-binary network on *X* and $\sigma _i :V(N_i) \rightarrow V(N)$ defines the origin of a node from $N_i$. Let $(N_0,\sigma _0)$ be a pair such that $N_0=N$ and $\sigma _0(v)=v$ for each $v \in V(N)$. Then, for a sequence of all reticulation nodes $r_1, r_2, \dots , r_k$ from *N* in a reversed topological order, $(N_i,\sigma _i)$ is obtained from $(N_{i-1},\sigma _{i-1})$ by unfolding the reticulation $r_i$ as follows:

- Let $S_i$ be a copy of the subtree of $N_{i-1}$ rooted at $r_i$.
- $V(N_{i}):=V(N_{i-1}) \cup V(S_i)$ and $E(N_{i}):=(E(N_{i-1}) \setminus \{ \langle p,r_i \rangle \}) \cup E(S_i) \cup \{ \langle p,r_i' \rangle \}$, where *p* is an arbitrary parent of $r_i$ and $r_i'$ is the root of $S_i$.
- $\sigma _i(v)$ is $\sigma _{i-1}(v)$ if $v \in V(N_{i-1})$; otherwise, it is $\sigma _{i-1}(t)$, if *v* is a copy of *t* from $N_{i-1}$.

**Fig. 2:**
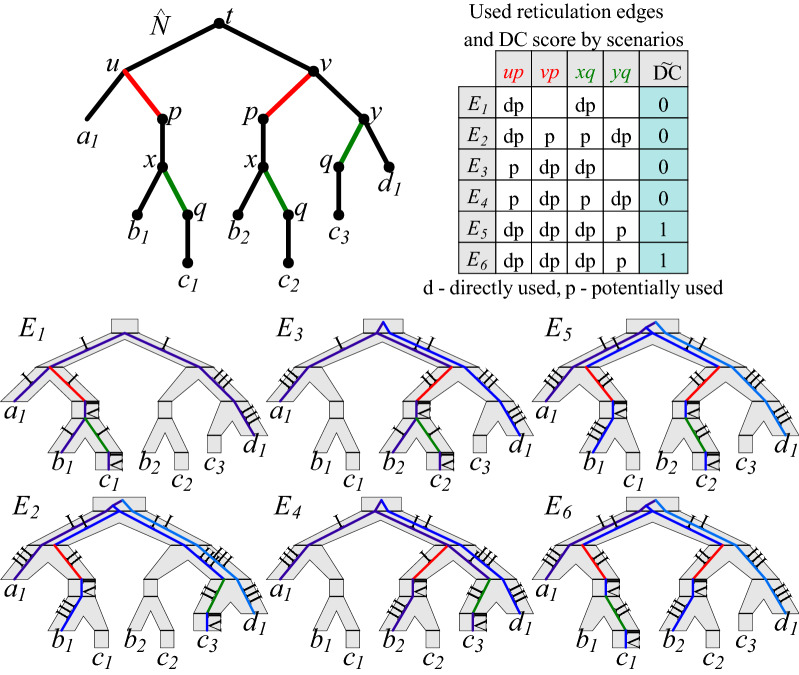
Top left: The unfolded network $\hat{N}$ of *N* from Fig. 1 is shown with $\sigma$ values attached to nodes, where for the leaves, the index is inserted to distinguish leaves with the same labels/mappings. Bottom: 6 scenarios for $G=((a,(b,c)),d)$ shown as embeddings of *G* to $\hat{N}$. Numbers I–IV denote the type of a visited edge. Only $E_1$ is regular, while $E_1-E_4$ are optimal. Top right: DC score and types of used reticulation edges for each scenario ($\Upsilon _{[.]}$).

Informally, for each reticulation node, we copy its subtree, detach the original subtree from one parent, and attach the copy to the same parent, without changing the labels. To avoid using *k* directly, we set $\sigma$ to be $\sigma _k$.^2^ Figure 2 depicts an unfolded network.

#### Lemma 2

*(Correctness of unfolding) The unfolded network* $\hat{N}$* of* *N is a semi-binary tree*.

#### Proof

The proof follows by induction on $i=0,1,\dots ,k$, by showing that $N_{i}$ is a semi-binary network on *X* with the reticulation nodes $r_{i+1},\dots ,r_{k}$ such that there is no reticulation node below $r_{i+1}$ in $N_i$. For $i=0$ the above statement holds trivially. For each $i>0$, $N_{i}$ is obtained from $N_{i-1}$ by unfolding $r_i$. Next, it follows from the topological order, and the inductive assumption, that there is no reticulation below $r_{i}$, thus the set of all nodes below $r_{i}$ induces a rooted subtree in $N_{i-1}$. $\square$

Let a *root-leaf* path be a directed path connecting the root with a leaf in a network.

#### Theorem 3

*(Unfolding Soundness) There is a one-to-one correspondence between root-leaf paths in* *N* *and root-leaf paths in* $\hat{N}$.

#### Proof

The bijection is established by $\sigma$, i.e., if $P=p_1,p_2,\dots ,p_m$ is a root-leaf path in $\hat{N}$, then $\sigma (P)=\sigma (p_1),\sigma (p_2),\dots ,\sigma (p_m)$ is the corresponding root-leaf path in the network *N*. $\square$

It follows from Theorem 3 that *N* and $\hat{N}$ have the same structure of root-leaf paths. *A scenario* for *G* and *N* is a function $\xi :L(G) \rightarrow L(\hat{N})$ that preserves the leaf labeling: for every $g \in L(G)$, the labels of *g* and $\xi (g)$ are equal. A scenario $\xi$ can be extended to the lca-mapping $\mathsf {M}_\xi :V(G) \rightarrow V(\hat{N})$ such that for $g \in V(G)$, $\mathsf {M}_\xi (g)$ is the lowest node *v* in $\hat{N}$ such that $\xi (g') \preceq v$, for each leaf $g' \preceq g$. Note that $M_\xi (g)$ is either a leaf or a tree node.

### Deep coalescence score of scenarios

Having the lca-mapping determined by a scenario, we are ready to define the deep coalescence *score*, denoted $\tilde{\mathsf {DC}}$, to approximate deep coalescence events induced by scenarios in phylogenetic networks. Our first goal is to deduce properties allowing us to approximate the DC cost to solve ODT-DC in the class of tree-child networks. In particular, our approach differs from the approaches from [7, 9, 10], e.g., in the way in which a cost of a path is defined, although the general concept of mapping a gene tree into a network is analogous.

For a scenario $\xi$ for *G* and *N*, we say that $\langle v,w \rangle \in E(G)$ *visits* $\langle a,b \rangle \in E(\hat{N})$ if $\mathsf {M}_\xi (v) \succeq a \succ b \succeq \mathsf {M}_\xi (w)$. Then, $\langle a,b \rangle$ has exactly one of the following *types*.

- Type I: $\mathsf {M}_\xi (v)=a$, i.e., it is the first edge.
- Type II: $\mathsf {M}_\xi (v) \succ a$, $\deg_{\hat{N}}(a)=2$ and $\sigma (b.{\mathsf {sibling}}) \notin R(N)$.
- Type III: $\mathsf {M}_\xi (v) \succ a$, $\deg _{\hat{N}}(a)=2$ and $\sigma (b.{\mathsf {sibling}}) \in R(N)$; we say that $\xi$ *bypasses* the reticulation edge $\sigma (\langle a,b.{\mathsf {sibling}} \rangle$).
- Type IV: $\deg _{\hat{N}}(a)=1$ (only if $\sigma (a) \in R(N)$).

In the above definition, type (I) is only for the first (i.e., the closest to the root) edge visited by a given edge from *G*, while for the remaining visited edges from $\hat{N}$ an edge has: Type (II) if the sibling of its bottom node is a tree node, Type (III) if the sibling of its bottom node is a reticulation, and Type (IV) if the top node of the edge is a reticulation.

By $\kappa _\xi (v,w)$ we denote the set of all edges of Type I or II visited by $\langle v,w \rangle$. Then, the *deep coalescence score* for *G*, *N* and a scenario $\xi$ is

2 $$\begin{aligned} \tilde{\mathsf {DC}}(G,N,\xi )=\sum _{\langle v,w \rangle \in E(G)} (|\kappa _\xi (v,w)|-1). \end{aligned}$$

Examples of scenarios and $\tilde{\mathsf {DC}}$ scores are depicted in Figure 2. Finally, we can define *Optimal Scenario Inference* problem, DC-MinRec.

#### Problem 4

(DC-MinRec) Given a gene tree *G* and a phylogenetic network *N*. Find an optimal scenario $\xi ^*$ that minimizes $\tilde{\mathsf {DC}}(G,N,\xi )$ in the set of all scenarios $\xi$ for *G* and *N*.

In the next sections, we propose a dynamic programming algorithm that solves DC-MinRec in *O*(|*G*||*N*|) time where *N* is a tree-child network. Note that the time complexity depends on the size of *N* (not on the potentially exponential size of $\hat{N}$).

In a trivial case, the solution to DC-MinRec is induced by the classical $\mathsf {DC}$ cost.

#### Lemma 5

*If* *N is a phylogenetic network with no reticulation node, there is only one scenario* $\xi$* for* *G and* *N*.* Moreover,* $\tilde{\mathsf {DC}}(G,N,\xi )=\mathsf {DC}(G,N)$.

#### Proof

*N* is a species tree and the scenario is determined by $\xi :=\mathsf {M}|_{L(N)}$. In this case, $\mathsf {M}_\xi =\mathsf {M}$, all visited edges in $\hat{N}$ are of Type I or II. Thus, $|\kappa _\xi (v,w)|=||\mathsf {M}(v),\mathsf {M}(w)||$ and the proof is straightforward from (1) and (2). $\square$

### Displayed trees in tree-child networks

Here, we present several important properties of displayed trees in tree-child networks.

Given a tree-child network *N* on *X*, a set $Y \subseteq E_R(N)$ is called *perfect* if, for each $r \in R(N)$, *Y* contains exactly one edge whose bottom node is *r*. Given a perfect *Y*, the graph denoted $N\setminus Y$, obtained from *N* by removing all edges from $E_R(N) \setminus Y$ is a semi-binary tree on *X*, i.e., semi-binary network with no reticulations.

#### Lemma 6

*Let* *N be a tree-child network on* *X and* $Y \subseteq E_R(N)$.* Then,* *Y is perfect if and only if* $N \setminus Y$* is a semi-binary tree on* *X*.

#### Proof

($\Rightarrow$) In a tree-child network a node cannot have all descendands being reticulations. Therefore, $N \setminus Y$ contains no unlabelled leaf. Next, every reticulation node *r* from *N* has exactly one parent in $N \setminus Y$. Also, $N \setminus Y$ is a connected graph on *X*, which follows by showing that each node is connected with the root. We omit easy inductive proof. ($\Leftarrow$) Let $r \in R(N)$. Then, *Y* must contain exactly one reticulation edge whose bottom node is *r*. Otherwise, $N \setminus Y$ is not a tree. $\square$

Since $N \setminus Y$ is a semi-binary tree on *X*, contracting all semi-binary nodes from $N \setminus Y$ yields a species tree $N_Y$ on *X*. Next, the subgraph $N \setminus Y$ of *N* is a subdivision of a tree $N_Y$ on *X*. We conclude that $N_Y$ is a displayed tree of *N*. We also have the following property.

#### Lemma 7

*Let* *T be a displayed tree of a tree-child network* *N*.* Then, there is a perfect set* *Y such that* $N_Y=T$.

#### Proof

Let $T'$ be a subgraph of *N* such that $T'$ is a subdivision of *T*. Let $Y=E_R(N) \setminus E(T')$. It remains to show that *Y* is perfect. Note that $T'$ is a semi-binary tree on *X* and the rest follows similarly to the case $(\Leftarrow )$ from Lemma 6. $\square$

We say that the perfect set *Y* is induced by a tree *T* displayed by *N* if $N_Y=T$. Note that different perfect sets may induce the same displayed tree. E.g., a tree child network with one reticulation and two leaves has two perfect sets each one inducing the same displayed tree.

Note that in more general cases of networks (see relaxed networks in Section *Beyond tree-child networks*) additional removal of non-labeled vertices with out-degree zero (i.e., unlabelled leaves) from $N \setminus Y$ is required to obtain a semi-binary tree on *X*.

### DC scores of scenarios vs. DC costs of displayed trees

This section presents several theoretical results connecting our scoring functions in the class of tree-child networks. Note that the notion of a *cost* will be used only with the $\mathsf {DC}$ cost defined in (1) for trees and for phylogenetic networks in Problem 1, while for scenarios, we will use the notion of a $(\tilde{\mathsf {DC}})$ *score*.

To establish the correspondence between DC scores and DC costs, we first show that each perfect set *Y* determines a scenario. Recall that $N_Y$ is obtained from N\Y by contracting semi-binary nodes. Let $\hat{N}_Y$ be the graph obtained from $\hat{N}$ by removing all edges *e* such that $\sigma (e) \in E_R(N)\setminus Y$ and all subtrees whose root is the bottom node of *e*.

#### Lemma 8

*Let* *N be a tree-child network and* $Y \subseteq E_R(N)$* be perfect. Then,* $\hat{N}_Y$* and* *N\Y are isomorphic, and the isomorphism is established by* $\sigma |_{V(\hat{N}_Y)}$.

#### Proof

The proof is by induction with unfolding steps. Using the same notation, we construct *N*/*Y* iteratively using the sequence of reticulation nodes from the construction of $\hat{N}$. Let $B_0=N$. For each $i=0,1,\dots ,k$, $B_i$ is inferred from $B_{i-1}$ by removing the reticulation edge from $E_R(N)\setminus Y$ adjacent to $r_i$. It is not difficult to see that $B_k=N \setminus Y$ (as we removed only edges from $E_R(N)\setminus Y$). $\hat{N}_Y$ can be equivalently obtained by modification of the original unfolding step by removing the copy $S_i$ or the original subtree rooted at $r_i$ depending on whether the corresponding reticulation edge is in *Y*. $\square$

For a tree-child network *N*, a gene tree *G* and a perfect set $Y \subseteq E_R(N)$, we define a scenario $\xi _Y$ for *G* and *N*, such that for each gene leaf *g* labeled *x*, $\xi _Y(g)$ is the only leaf in $L(\hat{N}_Y) \subseteq L(\hat{N})$ labeled *x*. Correctness follows from Lemma 8. For example, in Fig. 2, if $Y=\{ \langle u,p \rangle , \langle x,q \rangle \}$, then *Y* is perfect and $N_Y=S_1$ from Figure 1. Moreover, for $G=((a,(b,c)),d)$, $\xi _Y$ maps *a* to $a_1$, *b* to $b_1$ *c* to $c_1$ and *d* to $d_1$ as depicted in $E_1$.

We say that $e \in E_R(N)$ is *directly used* by scenario $\xi$ if there is a visited edge $e'$ of Type I or II such that $\sigma (e')=e$. Similarly, we say that reticulation edge *e* is *potentially used* by $\xi$ if the sibling edge of *e* is bypassed by $\xi$. By $\Upsilon _\xi \subseteq E_R(N)$ we denote the set of reticulation edges used directly or potentially by $\xi$ (see  Fig. 2).

We say that $Y \subseteq E_R(N)$ has a *conflict* if *Y* contains two sibling edges. We say that $\xi$ is *regular* if $\Upsilon _\xi$ has no conflict. For instance, $\Upsilon _{E_4}$ for $E_4$ from Fig. 2 has two possible conflicts in *N*. Observe that $\Upsilon _\xi$ may not be perfect in general, even if $\xi$ is regular. For instance, if $G=(c,d)$ and $\xi$ maps *c* to $c_3$ in the network from Fig. 2, then $\Upsilon _{\xi }=\{ \langle y,q \rangle \}$.

Now, we can state the crucial proposition that establishes a correspondence between regular scenarios and embedding to trees displayed by a tree-child network.

#### Proposition 9

*(Scenario-Displayed Tree Correspondence) Let* *N be a tree-child network and let* *G be a gene tree. A scenario* $\xi$* for* *G and* *N is regular, if and only if for every perfect set* *Y such that* $\Upsilon _\xi \subseteq Y$, $\tilde{\mathsf {DC}}(G,N,\xi )=\mathsf {DC}(G,N_Y)$.

#### Proof

($\Leftarrow$.) If $\Upsilon _\xi$ is a subset of a perfect set *Y*, then $\Upsilon _\xi$ has no conflict. Thus, $\xi$ is regular. ($\Rightarrow$). If $\xi$ is regular, then there is at least one perfect *Y* such that $\Upsilon _\xi \subseteq Y$. Based on the definitions of $\mathsf {DC}$ and $\tilde{\mathsf {DC}}$, it is sufficient to prove

3 $$\begin{aligned} |\kappa _\xi (v,w)|=||\mathsf {M}(v),\mathsf {M}(w)||, \end{aligned}$$

for every edge $\langle v,w \rangle \in E(G)$, where $\mathsf {M}$ is the lca-mapping between *G* and the species tree $N_Y$, for one fixed perfect set $Y \supseteq \Upsilon _\xi$.

We have $V(N/Y)=V(N) \supseteq V(N_Y)$, and $\mathsf {M}(g) \in V(N_Y)$ is a leaf or a tree node. Let $d=||\mathsf {M}(v),\mathsf {M}(w)||$ (in $N_Y$). Note that no removed edge from $\hat{N}$ is visited by scenario $\xi$, we conclude that $\sigma (\mathsf {M}_\xi (g))=\mathsf {M}(g)$ for every *g*. If $\mathsf {M}(w)=\mathsf {M}(v)$ then $|\kappa _\xi (v,w)|=d=0$. Otherwise, assume $\mathsf {M}(v) \succ \mathsf {M}(w)$. Let $P=p_1,p_2,\dots ,p_m$ be the directed path from $\mathsf {M}_\xi (v)$ to $\mathsf {M}_\xi (w)$ in $\hat{N}_Y$ (and in $\hat{N}$). Then, by Lemma 8, $\sigma (P)$ is the unique directed path in *N*/*Y* from $\mathsf {M}(v)$ to $\mathsf {M}(w)$. Note that *d* equals one plus the number of nodes of outdegree 2 located strictly between $\mathsf {M}(v)$ and $\mathsf {M}(w)$ in $N_Y$. The same statement holds in *N*/*Y*. We show that *d* equals the number of Type I and II edges in $\hat{N}$. For an edge $e_i=\langle p_i,p_{i+1} \rangle$, with $0<i<m$ we have, the following types of edges:

- Type I: the edge exists since $m>1$.
- Type II: $$\deg _{\hat{N}}(p_i)=\deg _{\hat{N}_Y}(p_i)=2$$.
- Type III: $\deg _{\hat{N}}(p_i)=2$ and $e_i$ bypasses the reticulation edge $e'=\sigma (\langle p_i,p_{i+1}.{\mathsf {sibling}}) \rangle )$. The sibling of $e'$ is in $\Upsilon _\xi \subseteq Y$. *Y* is perfect, so $e' \in E_R(N)\setminus Y$. Thus, $\deg _{\hat{N}_Y}(p_i)=1$.
- Type IV: $$\deg _{\hat{N}}(p_i)=\deg _{\hat{N}_Y}(p_i)=1$$.

As $\deg_{\hat{N}_Y}(p_i)=\deg_{N/Y}(\sigma (p_i))$, we see that $\deg _{N/Y}(\sigma (p_i))=2$ if and only if $i>1$ and $e_i$ has Type II. Moreover, the directed path contains one edge of Type I. Thus, $|\kappa _\xi (v,w)|=d$. This completes the proof of (3) and ($\Rightarrow$) implication. $\square$

In the following proposition, we show that the cost of a tree displayed by a network using a perfect set is bounded from below by the cost of its corresponding scenario.

#### Proposition 10

*Let* *N be a tree-child network and let* *G be a gene tree. If* $Y \subseteq E_R(N)$* is perfect, then* $\mathsf {DC}(G,N_Y) \ge \tilde{\mathsf {DC}}(G,N,\xi _Y)$.

#### Proof

The proof is similar to the proof of Proposition 9, with the difference that we show $||\mathsf {M}(v),\mathsf {M}(w)|| \ge |\kappa _{\xi _Y}(v,w)|$, for any gene tree edge $\langle v,w \rangle$, where $\mathsf {M}$ is the lca-mapping between *G* and $N_Y$. The only difference is in the edges of Type III in the last part of the proof. Here, we have $\deg _{\hat{N}}(p_i)=2$ and $e_i$ bypasses the reticulation edge $e'$. As we do not have the assumption that $Y_\xi \subseteq Y$, $e'$ may be present in *Y*. In such a case, $\deg _{\hat{N}}(p_i)=2$. Thus, the node $\sigma (p_i)$ has outdegree 2 in $\hat{N}_Y$. We conclude that $||\mathsf {M}(v),\mathsf {M}(w)||-|\kappa _{\xi _Y}(v,w)|$ is the number of edges of Type III on the directed path from $\mathsf {M}_\xi (v)$ to $\mathsf {M}_\xi (w)$ that bypass an edge from *Y*. This completes the first part of the proof. $\square$

Finally, we show that the equality between the score and the cost holds only if the induced scenario is regular.

#### Proposition 11

*Let* *N be a tree-child network and let* *G be a gene tree. If* $Y \subseteq E_R(N)$* is perfect, then* $\mathsf {DC}(G,N_Y) = \tilde{\mathsf {DC}}(G,N,\xi _Y)$* if and only if* $\xi _Y$* is regular*.

#### Proof

($\Leftarrow$). It follows from Proposition 9. ($\Rightarrow$). From the proof of Proposition 10, we conclude that equality holds only if there is no edge in *Y* bypassed by $\xi _Y$. Thus, each edge potentially used by $\xi _Y$ must be in *Y*. As every directly used edge is also in *Y*, by the construction of $\xi _Y$, we have $\Upsilon _\xi \subseteq Y$. Thus, $\Upsilon _\xi$ is regular. $\square$

The next theorem states that the cost of an optimal tree displayed by a network is bounded from below by the score of an optimal scenario.

#### Theorem 12

*(Lower Bound Property) Let* *N be a tree-child network and let* *G be a gene tree. If *$S^*$* is an optimal tree displayed by* *N*,* and* $\xi ^*$* is an optimal scenario for* *G and* *N then* $\mathsf {DC}(G,S^*) \ge \tilde{\mathsf {DC}}(G,N,\xi ^*)$.

#### Proof

If $S^*$ is a tree displayed by *N* then there is a perfect *Y* such that $S^*=N_Y$. Thus, we have $\mathsf {DC}(G,N_Y) \ge \tilde{\mathsf {DC}}(G,N,\xi _Y)$ from Proposition 10 and $\tilde{\mathsf {DC}}(G,N,\xi _Y) \ge \tilde{\mathsf {DC}}(G,N,\xi ^*)$ from the definition of $\xi ^*$. $\square$

In our example from Figs. 1 and 2, the cost of $S_1$ and the score of $E_1$ are equal. However, in general, a regular scenario may not exist. For instance, if $G=(a,d)$, there is only one scenario $\xi$ for *N* from Fig. 2, where *a* and *d* are mapped to $a_1$ and $d_1$, respectively. Then, $\xi$ is not regular, and $0=\tilde{\mathsf {DC}}(G,N,\xi )<\mathsf {DC}(G,N)=1$ (for $S_1$ or $S_2$).

Finally, we present a crucial theoretical property used to solve ODT-DC in class of tree-child networks using solutions to instances of DC-MinRec.

#### Theorem 13

*(Regularity) Let* *d be the score of an optimal scenario of a gene tree* *G and a tree-child network* *N*.* A tree* *S displayed by* *N with* $\mathsf {DC}(G,S)=d$* exists, if and only if there is an optimal regular scenario of* *G and* *N*.

#### Proof

($\Leftarrow$). Take any perfect set *Y* such that $Y_{\xi ^*} \subseteq Y$ and $S:=N_Y$. The equality follows from Proposition 11. ($\Rightarrow$). By Theorem 12, *S* is an optimal tree displayed by *N*, since *d* is a lower bound for the cost of a displayed tree. Now, we take the perfect set *Y* induced by *S*. The scenario $\xi _Y$ has score *d*. Hence, it is optimal. By Proposition 11, $\xi _Y$ is also regular. $\square$

### Dynamic programming (DP) algorithms to solve DC-MinRec

Dynamic programming algorithms are commonly used in tree reconciliation, including models based on directed acyclic graphs (DAGs) [7, 9, 17, 23, 27], where a gene tree is mapped to a tree or a DAG through the lca-mapping or general mapping based on concepts close to our scenarios. Such approaches often lead to polynomial time solutions with square time complexity in the best case. Here, we present two dynamic programming solutions to Problem 4 by providing formulas to compute the score of an optimal scenario. We start with a simplified and computationally demanding DP formulation. Then, we show an efficient approach running in square time.

*Additional notation:* By $v'$ and $v''$, we denote the children of $v \in T(N)$, and by $r'$ the child of a reticulation node *r*. For simplicity, instead of $\sigma (M_\xi (g))$ for a gene tree node *g*, we write $\xi _g$ (i.e., $\xi _{[.]}$ is a mapping from *G* to *N*).

#### Dynamic programming formulation in $O(|G||N^3|)$ time: the first approach

We can express the formula for $\delta$ in the following way:

4 $$\begin{aligned} \delta(g,s):={\left\{ \begin{array}{ll} \min _{s \succeq t,u} \delta (g',t)+\pi (s,t)+\delta (g'',u)+\pi (s,u) &{} g \in T(G)\text{ and }s \notin R(N), \\ 0 &{} g, s\ \text{are leaves with the same label,}\\ +\infty &{} \text {otherwise}, \end{array}\right. } \end{aligned}$$

where, for $s \succeq t$ in *N*,

5 $$\begin{aligned} \pi(s,t):={\left\{ \begin{array}{ll} ||s,t|| &{} ||s,t|| \le 1\ \ \text{(the empty path or Type I)}, \\ 1+\pi (s,t.\mathsf {parent}) &{} \{t, t.{\mathsf {sibling}}, t.\mathsf {parent}\} \cap R(N) = \emptyset \ \ \text{(Tp. II)}, \\ 1 + \min (\pi (s,\dot{t}),\pi (s,\ddot{t})) &{} t \in R(N)\ \ \text{(Type II)}, \\ \pi (s,t.\mathsf {parent}) &{} t.{\mathsf {sibling}} \text{ or } t.\mathsf {parent}\in R(N)\ \text{(Type III/IV)}, \\ +\infty &{}\text{otherwise.}\end{array}\right. } \end{aligned}$$

Here $\dot{r}$ and $\ddot{r}$ denote the parents of a reticulation node *r*.

The correctness of the above formulas follows from the following two Lemmas.

##### Lemma 14

*(Correctness of* $\pi$*) For* $s \succeq t$, $\pi (s,t)$ *is the minimal number of Type I or II edges between two nodes* $a \succeq b$* in* $\hat{N}$* such that* $\sigma (a)=s$* and* $\sigma (b)=t$.

##### Proof

It follows from Theorem 3, that $\pi$ can be computed directly from *N*. The proof is by induction on the length of the directed path. The cases in $\pi$ formulas correspond directly to the types of edges (see comments in (5)), where we add/set 1 if the visited edge has Type I or II. Note that there is only one branching when $t \in R(N)$. In such a case, the formula will choose the directed path to *s* with the lower cost. We omit technical details. $\square$

##### Lemma 15

*(Correctness of* $\delta$*) For g* *and* *s*, $\delta (g,s)$ *from Equation (*4) *is the minimal number of Type I/II edges visited by edges from* *E*(*G*|*g*)* in a scenario* $\xi$,* in the set of all scenarios *$\xi$* between G and* *N such that* $\xi _g=s$.

##### Proof

The proof follows by the induction on the structure of *G* and *N*, where Lemma 14 is applied to prove the induction hypothesis in each step. We omit easy details. $\square$

Computing $\delta$ using the above formulas requires $O(|N|^2+|G||N|^3)$ time and $O(|G||N|+|N|^2)$ space. Therefore, this approach is rather prohibitive for larger instances.

#### Efficient DP solution in *O*(|*G*||*N*|) time

By *G*|*g*, we denote the subtree of *G* rooted at *g*. The main component of dynamic programming is $\delta$ such that for $g \in V(G)$ and $s \in V(N)$, $\delta (g,s)$ is the minimum score for *G*|*g* in the set of all scenarios $\xi$ between *G*|*g* and $\hat{N}$ such that $\xi _g=s$. For simplicity, we ignore $-1$ from the $\tilde{\mathsf {DC}}$ formula in the partial costs in $\delta$ as this yields a constant term dependent on the size of *G*.

Let $\tau (s)$ be 0 if *s* is a reticulation, and 1 otherwise. Then, we have the following dynamic programming formula that solves DC-MinRec: 
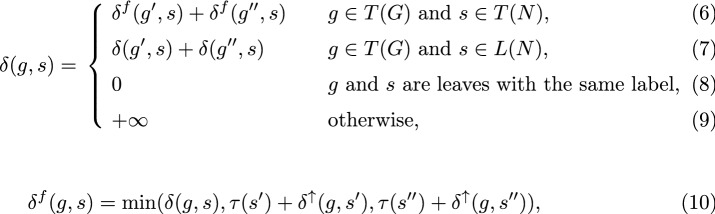

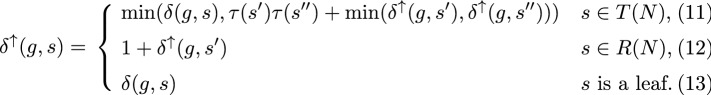


In the next Lemma, we express properties satisfied by the above formulas.

##### Lemma 16

*Let* $g \in V(G)$, $s \in V(N)$* and all scenarios below are for* *G and* *N*.

D1: $\delta (g,s)$ is equal to minimum number of Type I/II edges visited by edges from *E*(*G*|*g*) among scenarios $\xi$ satisfying $\xi _g=s$.
D2: If *c* is a child of *g* and *t* is not a reticulation. Then, $\delta ^\uparrow (c,t)$ is equal to minimum number of Type I/II edges visited by edges from *E*(*G*|*c*) plus the number of edges $e'=\langle a,b \rangle$ of Type II visited by $\{\langle c,g \rangle \}$ with $t \succeq \sigma (a)$ among scenarios $\xi$ such that $\xi _{g} = s \succ t \succeq \xi _c$.
D3: If *c* is a child of *g* and *s* is a tree node. Then, $\delta ^f(c,s)$ is equal to minimum number of Type I/II edges visited by edges from $E(G|c) \cup \{\langle c,g \rangle \}$ among scenarios $\xi$ satisfying $\xi _{g}=s$.

##### Proof

We follow with D1, where the proofs for D2-D3 are included as internal statements. The proof is by induction on the structure of *G* and *N*. The base is when *g* and *s* are leaves, for which D1 is obvious. *Inductive assumption:* D1 holds, for every *x*, *y* such that $g \succeq x$, $s \succeq y$, and either $x \ne g$ or $y \ne s$. *Inductive hypothesis:* D1 holds for *g* and *s*, where at least one of *g* and *s* is not a leaf.

*The proof for D2.* Let $\xi$ be the scenario having the minimal number of I/II-edges as defined in *D*2 and let *k* be the number of these edges. We follow by induction by assuming that $\delta ^\uparrow (c,u)$ satisfies D2 for all *u*, such that $t \succ u \notin R(N)$. We prove D2 for *t*. The base step is when $\xi _c=t$. Then, $k=\delta (c,t)$ and D2 follows from the induction assumption for D1 with cases (11) (the first expression when *t* is a tree node) and  (13) (when *t* is a leaf). Assume that $t \succ \xi _c$, then $\langle g,c \rangle$ visits the edge $e=\langle a,b \rangle$ such that $\sigma (e)=\langle t,t' \rangle$. We have three cases. (Case D2.i) If both children of *t* are tree nodes, then *e* has Type II. Note that $\xi$ also has the minimal number of edges satisfying the inductive assumption with nodes *c* and $t'$. Otherwise, if there is a scenario $\xi '$ with a score $<k-1$ for *c* and $t'$ then by visiting *e*, we will have a scenario with $<k$ edges for D2. This contradicts the assumption that *k* is minimal. Thus, $k=1+\delta ^\uparrow (c,t')$, by the inductive assumption. As $\tau (t')\tau (t'')=1$, this matches the second expression in  (11). (Case D2.ii) If $t'$ is a tree node and $t''$ is a reticulation then $\xi$ bypasses the reticulation edge $\langle t,t'' \rangle$. Similarly to the previous case, we show that $\xi$ satisfies inductive assumption with *c* and $t'$ (we omit details). Thus, $k=\delta ^\uparrow (c,t')$ and $\tau (t')\tau (t'')=0$, again this matches the second expression in  (11). (Case D2.iii) If $t'$ is a tree node and $t'$ is a reticulation then $\xi$ directly uses reticulation edge $\langle t,t' \rangle$, i.e., *e* has Type II. Again, we show that $\xi$ satisfies inductive assumption with *c* and a tree node *v* being the child of reticulation $t'$ (we omit details). By the inductive assumption, we have $k=1+\delta ^\uparrow (c,v)$, which equals $\delta ^\uparrow (c,t')=1+\delta ^\uparrow (c,v)$, by  (11) with $\tau (t')\tau (t'')=0$, then by (12).

*The proof for D3.* Let $\xi$ be the scenario having the minimal number of I/II-edges as defined in *D*3 and let *k* be the number of these edges. If $\xi _c=s$, then there is no edge visited by $\langle c,g \rangle$. Thus, $k=\delta (c,s)$ by the induction assumption, which is the first expression in $\min$ of (6). Otherwise, assume that for a child $s'$ of *s*, $s' \succeq \xi _c=t$. Then, there is one edge of Type I visited by $\langle c,g \rangle$. We have two cases. (Case D3.i) If $s'$ is a reticulation, then $\tau (s')=0$ and $k=\delta ^\uparrow (g,s')=1+\delta ^\uparrow (g,t)$ where *t* is the child of $s'$. The latter follows from (12) and D2 (with $s:=t$). Note that $\xi$ has the minimal number of edges $k-1$ satisfying the corresponding assumptions of D2 (see a similar argument in the proof of case D2.i). (Case D3.ii) If $s'$ is a tree node or a leaf then $k=1+\delta ^\uparrow (g,s')$ by D2 (with $s'$). In both cases Type I edge is included. The rest is similar to case D3.i. This completes the proof of D3.

*The proof of D1.* It follows from D3, that $\delta ^f(c,s)=\min _{s\succeq t} \delta (c,t) + \pi (s,t))$ (see def. of $\pi$ in section Dynamic programming formulation in $O(|G||N^3|)$ time: the first approach), for a child *c* of *g*. Thus, if *g* and *s* are tree nodes we show that $\delta (g,s)=(\min _{s\succeq t} \delta (g',t) + \pi (s,t)) + (\min _{s\succeq u} \delta (g'',u) + \pi (s,u))$. The proof follows similarly to the previous cases by analysing $\xi$ with the minimal number of edges satisfying constraints from D1 (see also the recursion from (4) and Lemma 15). The case relates to (6). We skip details. Finally, we have two remaining cases. If *g* is a leaf and *s* is a tree node, then there is no scenario $\xi$ satisfying $\xi _g=s$. Then, the number is $+\infty$ (case (9)). If *s* is a leaf and *g* is a tree node, we have $\delta (g,s)=0$ if all leaves below *g* are labeled by the label of *s*, and $+\infty$ otherwise. This agrees with the number of visited Type I/II edges, where, in the second case, the set of scenarios satisfying the assumptions is empty. $\square$

The optimal score is given by the following theorem, whose proof follows immediately from the definitions of $\delta$, $\tilde{\mathsf {DC}}$ and Lemma 16.

##### Theorem 17

*Given a gene tree* *G and a tree-child network* *N*.* The score of an optimal scenario* $\xi ^*$* is* $\tilde{\mathsf {DC}}(G,N,\xi ^*) = -|E(G)| + \min _{s \in V(N)} \delta (G.{{\,\mathrm{\mathsf {root}}\,}},s).$

To infer an optimal scenario, we apply standard backtracking based on values of $\delta$ array. Since there are three arrays, each of size |*G*||*N*| and every cell of an array can be computed in *O*(1) time, DP has *O*(|*G*||*N*|) time and space complexity. Note that in implementation $\delta ^f$ can be embedded into $\delta$ computation. Thus, the space may be reduced to two arrays.

### Inferring used reticulations edges from DP

An optimal scenario can be inferred from DP formulas using standard backtracking. However, this scenario may not be perfect. To further utilize the results of DP, we infer the set of used reticulation edges. For two nodes *v* and *w*, let $\rho (v,w)=\{ \langle v,w \rangle \}$ denote the one-element set with $\langle v,w \rangle$ if this edge is a reticulation edge in *N*, and $\rho (v,w)=\emptyset$ otherwise. Similarly, by $\bar{\rho }(v,w)$ we denote the one-element set with the sibling edge of $e=\langle v,w \rangle$ if *e* is a reticulation edge in *N*, and $\bar{\rho }(v,w)=\emptyset$, otherwise. Then, DP components $\delta$, $\delta ^f$ and $\delta ^\uparrow$ are associated with reticulation edge usage rules *u*, $u^f$, and $u^\uparrow$, resp., as follows:

$$\begin{aligned} u(g,s)&={\left\{ \begin{array}{ll} u^f(g',s) \cup u^f(g'',s) &{} \text{in (6),} \\ \emptyset &{} \text{in (7)-(9),} \end{array}\right. }\\ u^f(g,s)&={\left\{ \begin{array}{ll} u(g,s) &{} \text{if}\ \delta ^f(g,s)=\delta (g,s)\ \text{in}\ (10),\\ u^\uparrow (g,c) \cup \rho (s,c) & \text{if}\ \delta^f(g,s)=\tau (c)+\delta^\uparrow(g,c)\ \text{for some}\ c \in \{s',s''\} \ \text{in}\ (10), \end{array}\right.}\\ u^\uparrow(g,s)&={\left\{ \begin{array}{ll} u(g,s) &{} \text{if}\ \delta ^\uparrow (g,s)=\delta (g,s)~\text{in}~(11)~\text{or}~(13),\\ u^\uparrow (g,c) &{}\text{in}~(12), \\ u^\uparrow (g,c) \cup \rho (s,c) \cup \bar{\rho }(s,c.{\mathsf {sibling}}) &{} \delta ^\uparrow (g,s)=\tau (s')\tau (s'')+\delta ^\uparrow (g,c) \\ &{} \text {for some}~c \in \{s',s''\}~\text{in}~(11). \end{array}\right. } \end{aligned}$$

The correctness of above formulas follows from the next lemma.

#### Lemma 18

*If the backtracking of DP results in a scenario* $\xi$,* then* $\Upsilon _\xi =u(G.{{\,\mathrm{\mathsf {root}}\,}},\xi _{G.{{\,\mathrm{\mathsf {root}}\,}}})$.

#### Proof

The proof follows by analysis of cases when reticulation edges are directly or potentially used by $\xi$ and it is based on the details from the proof of Lemma 16. There are three main cases when a reticulation edge is inserted using $\rho$ or $\bar{\rho }$.

Case I. When the first edge (Type I) on the visited, directed path is a reticulation edge, then, its corresponding reticulation edge from *N* is inserted in $u^f(g,s)$ using $\rho (s,c)$ in the second case. See also (D3.ii) in the proof of Lemma 16.

Case II. When the visited reticulation edge has Type II, then the corresponding reticulation edge is inserted in $u^\uparrow (g,s)$ using $\rho (s,c)$ in the last case. See also (D2.iii) in the proof of Lemma 16.

Case III. When the scenario bypasses a reticulation edge *e*, then *e* inserted in $u^\uparrow (g,s)$ using $\bar{\rho }(s,c.{\mathsf {sibling}})$ in the last case. See also (D2.ii) in the proof of Lemma 16. $\square$

### Inferring optimal displayed trees under deep coalescence cost

In this Section, we propose algorithms to solve ODT-DC in the class of tree-child networks. We also show how to adopt the solution to use structural properties of tree-child networks (e.g., level-*k* tree-child networks). Also, we answer whether the problem can be analogously solved when the class of networks is broader than tree-child.

#### Solution to ODT-DC in the class of tree-child networks

Theorem 13 motivates the following general branching algorithm to solve ODT-DC. Suppose DP returns a solution with a conflict. Then, such a conflict can be resolved by branching and solving two sub-instances of the problem with phylogenetic networks induced from the input phylogenetic network by removing exactly one edge from the conflict. Let $N_e$ be the tree-child network obtained from $N/\{e\}$ by contracting all semi-binary nodes^3^. Algorithm 1 details the procedure to infer an optimal tree displayed by a given network. Here, branching occurs when there is a conflict in the set of used reticulation edges. Thus, if the number of conflicts is low, e.g., when *G* and *N* are similar, we expect a small number of DP invocations. 
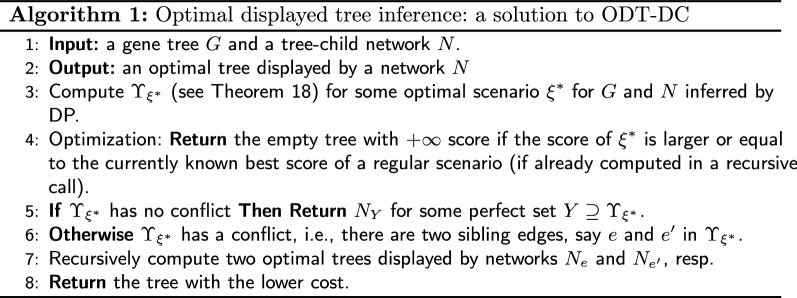


Correctness of Algorithm 1 follows from Theorem 13 and the following theorem.

##### Theorem 19

*If* *e*, $e' \in E_R(N)$* are sibling edges then* $\mathsf {DC}(G,N)=\min \{ \mathsf {DC}(G,N_{e}), \mathsf {DC}(G,N_{e'}) \}$.* Moreover,* *T is an optimal tree displayed by* *N if and only if T* *is an optimal tree displayed by a network* $N_e$* or* $N_{e'}$* with minimum cost*.

##### Proof

Let $\Delta (N)$ be the set of all trees displayed by *N*. Then, the first statement follows from the fact that for tree-child networks, $\Delta (N)=\Delta (N_e) \cup \Delta (N_{e'})$ and $\Delta (N_e) \cap \Delta (N_{e'})=\emptyset$. The second statement follows easily from the above observation. $\square$

In the worst case, we need to branch for every reticulation twice, which gives $2^{r+1}-1$ invocations of DP. Thus, Algorithm 1 has time complexity $O(2^r |G||N|)$ in the worst case. However, as mentioned previously, we expect Algorithm 1 to behave better than worst complexity in practice. See also our experimental evaluation in Section *Results*.

#### Lower and upper bounds of the optimal cost of a displayed tree

[3]Recall that N/X is the network obtained from N by removing all edges from X.
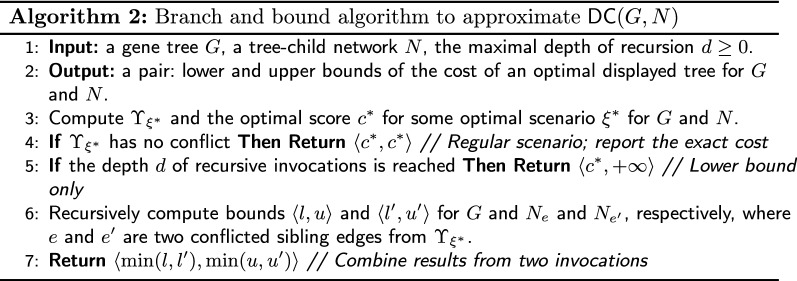


In applications where only the optimal cost is needed, for instance, in problems of network inference, we can use the Lower Bound Theorem 12. As the cost of an optimal displayed tree is bounded below by the score from DP, we can also compute the upper bound using regular scenarios returned from multiple invocations of DP. See details in Algorithm 2.

##### Lemma 20

*For a gene tree* *G and a tree-child network* *N*,* Algorithm 2 returns* *l and* *u such that* $l \le \mathsf {DC}(G,N) \le u$.

##### Proof

The proof follows by induction on the number of reticulation nodes in a network. If *N* is a tree, then the statement is obvious, as the scenario has no conflicts $l=u=DC(G,N)$. Otherwise, we have several cases. If the scenario from DP has no conflict, then we have the exact solution (see Line 4). Otherwise, there is a conflict, and if the recursion depth is reached, then the computation is completed in Line 5 with proper bounds (see Theorem 12). In the final case, we have two pairs of bounds from two invocations. By the inductive assumption, the bounds are correct for $N_e$ and $N_{e'}$. For the lower bound of *N*, we have to take a minimum of *l* and $l'$, as there may exist the optimal scenario for the network $N_e$ or $N_e'$ with the cost $\min (l,l')$ in the “worst” case. Such a scenario is optimal for *N*. Similarly, we proceed with the upper bound. $\square$

#### Inferring optimal trees displayed by level-k tree-child networks

Our results can also be extended to level-*k* tree-child networks. The definition and properties are adopted from [9, 39, 40]. A level-*k* network is a phylogenetic network in which every biconnected component has at most *k* reticulation nodes [39]. If *B* is a biconnected component of *N*, then by $B.{{\,\mathrm{\mathsf {root}}\,}}$ we denote the unique node in *B* with no ancestors in *B*. Using the notation from [9], by *bc*(*N*) we denote the tree obtained from *N* by contracting all its biconnected components. Let $\mathsf {Lab}(N)$ denote the set of species present in *N* as leaf labels. 
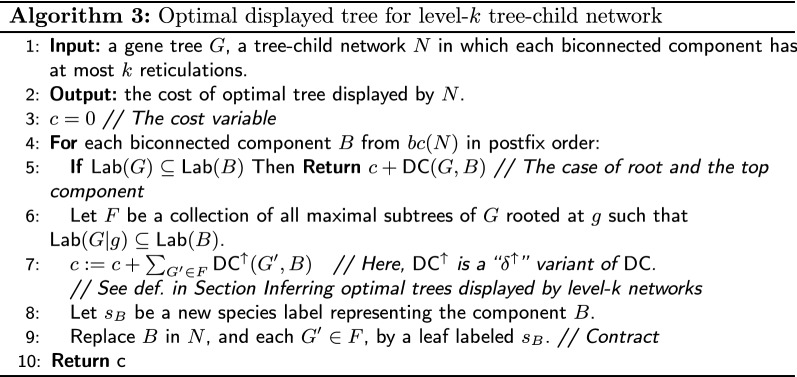


In Algorithm 3, edges visited by subtrees of *G* have to be connected in the embedding. Therefore, for each non-root component *B* in *b*(*N*), we minimize the score using the additional costs of a path to the root of *B*. Formally, ${\mathsf {DC}^{\uparrow }}(G,N)$ is the minimum value of $\mathsf {DC}(G,S)+||M(G.{{\,\mathrm{\mathsf {root}}\,}}),S.{{\,\mathrm{\mathsf {root}}\,}}||$ in the set of all displayed trees *S* of *N*. Computing the value (almost) does not require modification of our algorithms. Here, instead of the formula from Theorem 17, we compute ${\mathsf {DC}^{\uparrow }}(G,N)$ using $-|E(G)|+\delta ^\uparrow (G.{{\,\mathrm{\mathsf {root}}\,}},S.{{\,\mathrm{\mathsf {root}}\,}})$. The correctness follows from Lemma 16 case D2. The formula can be easily embedded into Algorithm 1. The time complexity of Algorithm 3 is $O(2^k|G||N|)$.

#### Beyond tree-child networks

**Fig. 3:**
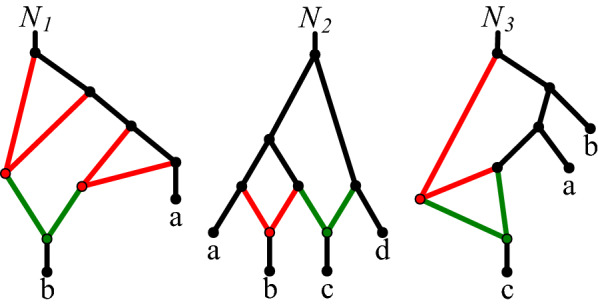
*Left:* A relaxed network $N_1$ which is not tree-sibling. *Middle:* A tree-sibling network $N_2$ which is not relaxed network. *Right:* A non tree-child network $N_3$ (also non-relaxed).

DP can be extended to analyse a broader class of networks, which is more beneficial from a practical point of view. Assume that instead of a tree-child network condition, our class of networks satisfies a relaxed condition: *each node has at most one reticulation child*. This assumption admits the child of reticulation to be a reticulation, which is not allowed in tree-child networks. Such networks, we call *relaxed*. We did not find an equivalent class in the literature. Note that the relaxed class is incomparable with a well-known class of Tree-Sibling networks (see networks $N_1$ and $N_2$ in Fig. 3), characterized by the condition: *each reticulation has a tree-node sibling*. Also, relaxed networks are not stable [41] in general, since the relaxed condition admits non-compressed networks (see Theorem 1 from [38]). For example, $N_1$ from Fig. 3 is not stable.

For the relaxed class, we modify DP in (12): $\tau (s')+\delta ^\uparrow (g,s')$, and in usage rules in the 2nd case of $u^\uparrow$ referring to (12): $u^\uparrow (g,s) \cup \rho (s,c)$, which is needed when the child is also a reticulation. Under this modification, Algorithm 1 returns a correct optimal displayed tree. We omit details for brevity.

We also analysed a general class of binary networks, i.e., in which a tree node may have two reticulation children. However, DP cannot correctly analyse such networks. When embedding a gene tree (*a*, *b*) into the network $N_3$ from Fig. 3, we see that the optimal displayed tree is $S=((a,b),c)$ with the cost 0. Here, *S* is constructed by removing a node *x* and all three incident edges, and a tree node $x.\mathsf {parent}$ with two children is also contracted. In the current DP, when a gene edge $\langle a.\mathsf {parent},a \rangle$ from *G* visits $x.\mathsf {parent}$ DP will increase the cost. Therefore, the lower bound property is not satisfied in this case unless a solution in which such removed tree nodes are detected is implemented. It remains open whether it can be done in polynomial time without checking all variants of displayed trees.

### Optimal displayed trees under gene duplication cost (ODT-DUP)

Algorithms presented in the previous Section can naturally by modified to operate on cost functions such as gene duplication or gene duplication and loss [16]. The main difference is the way an optimal scenario is computed. Here, we present a dynamic programming solution for optimal scenario problem under duplication cost. Since the results are analogous to the deep coalescence cost, we omit most of the theoretical details for brevity.

Given previously defined *lca-mapping* between a gene tree *G* and a species tree *S*, $\mathsf {M}:V(G) \rightarrow V(S)$, we define *duplication* contribution of vertex $g \in V(G)$, which has two children $g', g''$ as

14 $$\begin{aligned} \mathsf {dup}(g) = {\left\{ \begin{array}{ll} 1 &{} \text {if } \mathsf {M}(g) = \mathsf {M}(g') \text { or } \mathsf {M}(g) = \mathsf {M}(g'') \text {,}\\ 0 &{} \text {otherwise.} \end{array}\right. } \end{aligned}$$

If *g* is a leaf, then $\mathsf {dup}(g) = 0$. Then, the *duplication cost* between *G* and *S* (denoted by $\mathsf {DUP}(G, S)$) is defined as $\mathsf {DUP}(G, S) = \sum _{g \in T(G)} \mathsf {dup}(g)$ [22].

In this section we solve the following problem.

#### Problem 21

(DUP-ODT) Given a rooted tree *G* and a phylogenetic network *N*. Find a tree *S* displayed by *N* with the minimum $\mathsf {DUP}(G,S)$.

Similarly to deep coalescence score, given a scenario $\xi :V(G) \rightarrow V(S)$, we define *duplication score contribution* of a vertex $g \in V(G)$ as follows. If there is a child $g'$ of *g* such that $\mathsf {M}_\xi (g) = \mathsf {M}_\xi (g')$, then $\tilde{\mathsf {dup}}(g)=1$. Otherwise, $\tilde{\mathsf {dup}}(g)=0$. Then, the *duplication score* for *G*, *N* and a scenario $\xi$ is defined as $\tilde{\mathsf {DUP}}(G,N,\xi )=\sum _{g \in T(G)} \tilde{\mathsf {dup}}(g)$. Duplication score has analogous properties to the ones proved in Theorem 12 and Theorem 13, thus the score can be applied in our branch-and-bound framework. We omit details for brevity.

Similar to the DC case, we have two dynamic programming arrays, $\delta$ and $\delta ^\uparrow$. Recall, that $\delta (g,s)$ is the minimum score for *G*|*g* in the set of all scenarios $\xi$ between *G*|*g* and $\hat{N}$ such that $\xi _g=s$, and $\delta ^\uparrow (g, s)$ is the minimum score for *G*|*g* in the set of all scenarios $\xi$ between *G*|*g* and $\hat{N}$ such that $\xi _g=y$, where $s \succeq y$. Dynamic programming formulation is as follows 
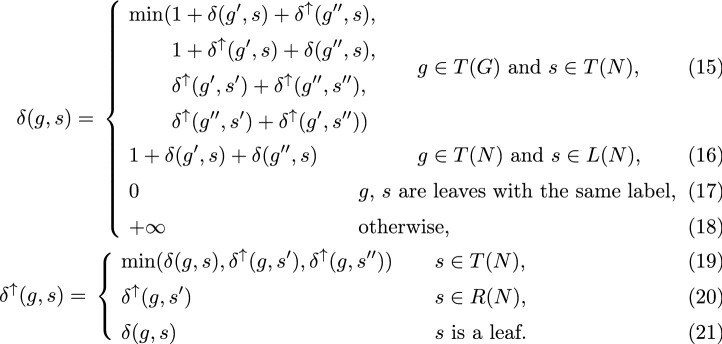


DP components $\delta$ and $\delta ^\uparrow$ are associated with usage rules *u*, $u^\uparrow$ respectively, as follows:

$$\begin{aligned} u(g,s)&={\left\{ \begin{array}{ll} u(g', s) \cup u^\uparrow (g'', s) &{} \text {for first case in (15),} \\ u^\uparrow (g', s) \cup u(g'', s) &{} \text {for second case in (15),} \\ u^\uparrow (g', s') \cup u^\uparrow (g'', s'') \cup \rho (s, s') \cup \rho (s, s'') &{} \text {for third case in (15),} \\ u^\uparrow (g'', s') \cup u^\uparrow (g', s'') \cup \rho (s, s') \cup \rho (s, s'') &{} \text {for fourth case in (15),} \\ \emptyset &{} \text {in (16)-(18),} \end{array}\right. }\\ u^\uparrow (g,s)&={\left\{ \begin{array}{ll} u(g,s) &{}\text{if}~\delta ^\uparrow (g,s)=\delta (g,s)~\text{in}~(19)~\text{or}~(21),\\ u^\uparrow (g,c) &{} \text {in (20),}\\ u^\uparrow (g,c) \cup \rho (s,c) &{} \text{if}~\delta ^\uparrow (g,s)=\delta ^\uparrow (g,c)~\text{for some c}~\in \{s',s''\}~\text{in}~(19). \end{array}\right. } \end{aligned}$$

## Results

In this section, we present the experimental evaluation using our prototype implementation of DP, Algorithm 1 and Algorithm 2 called EmbRetNet written in Python 3. The algorithms were extended to analyse the class of networks in which a node has at most one reticulation child (see discussion in the previous sections). The software package is available from the bitbucket repository.

### Performance of inferring optimal displayed trees

We show the performance of our algorithm where the gene tree is simulated using its network. Since both the gene tree and its network are topologically related, we expect better performance than in the worst case scenario. We summarize the results of several experiments to compare the performance of our implementation of Algorithm 1 to the naïve implementation in which all trees displayed by a given network are generated, and then, the costs are computed using a linear time solution from [15]. Note that both algorithms have exponential time complexity; however, the naïve algorithm always has the same number of steps, proportional to $2^r(|G|+|N|)$, where *r* is the number of reticulations in *N*. Experiments were conducted on a Ubuntu server with Intel(R) Xeon(R) CPU E5-2698 v4@2.20GHz (80 cores) and 500 GB of RAM.

#### Evaluation on random datasets

*Data preparation:* To generate random tree-child phylogenetic networks, we used an algorithm from [42] and its implementation in Python from GitHub with a slight modification to generate only binary networks. Random gene trees with one-to-one labeling of leaves were generated using the Yule-Harding model. Then, we generated datasets R1, R2, and R3, each consisting of $2 \cdot 10 \cdot 100$ pairs of random gene trees and networks. For each $n \in \{12,20\}$ and $r \in \{1,2,\dots ,10\}$, we generated 100 pairs $\langle G,N \rangle$, such that *N* is a network with *n* leaves and *r* reticulations and in dataset R1 $|L(G)|=n$, in R2 the number of leaves in *G* is sampled uniformly from the interval [2, *n*], and in R3 *G* is a randomly chosen tree displayed by *N*.

**Fig. 4:**
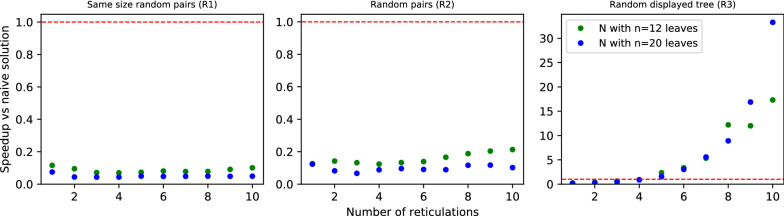
Performance of Algorithm 1 vs naïve approach for random datasets R1, R2 and R3. Each dot represents the average speedup computed from the runtimes of 100 pairs of gene trees and phylogenetic networks.

*Discussion:* In Figs. 4 and 6, we summarize the experimental evaluation results of R1, R2 and R3 with our solution to ODT-DC, where *G* and *N* were generated independently. Since R1 and R3 represent extremes, in which *G* and *N* are highly different, our algorithm frequently infers conflicted sets of reticulation edges by visiting almost every possible scenario, thus achieving nearly the pessimistic exponential complexity. In consequence, it is noticeably slower than the naïve one for R1. On the contrary, with data from R3 the algorithm rarely branches, and its average runtime matches the complexity of DP, i.e., *O*(|*G*||*N*|). Even with a larger constant factor, we outperform the naïve algorithm for $r > 4$, achieving $>15$ times speedup for $r = 10$.

### Evaluation on simulated datasets

Our simulation procedure can be divided into three major phases: (i) simulating tree-child phylogenetic networks, (ii) simulating gene trees, and (iii) introducing errors to the gene trees. The selection of the parameters in all three phases is mainly based on the simulation study conducted by Molloy and Warnow [43], which uses parameters derived from a fungal dataset presented by Rasmussen and Kellis [44].

*(i) Simulating tree-child phylogenetic networks.* First, we simulated species trees using a general sampling approach implemented in R package TreeSim version 2.4 [45] with the parameters from [43]. Specifically, we ran sim.bd.taxa.age function with the following parameters: tree height = 1800000337.5 years, speciation rate = $1.8*10^{-9}$ events/year and extinction rate = 0 events/year. The number of leaves was set to 12 or 20.

After simulating each species tree, we assigned a time value to all of its nodes, corresponding to a length of a path connecting the root with the node. Note that the general sampling approach produces ultrametric species trees, therefore time values assigned to the leaves were equal.

Next, we inferred a network with *k* reticulations from each of the simulated species trees, where *k* was uniformly sampled from [1, 10]. We added *k* reticulations one by one, following a popular study by Solis-Lemus and Ané [46]. Similarly to [8] we constrained the networks to a tree-child class. To add a reticulation edge to a species tree/network, we started by randomly choosing a pair of distinct non-reticulation edges and subdivided them, making two new vertices. We then sampled a time value for each of the vertices from uniform(vertex.parent.time, vertex.child.time). Finally, we added a reticulation edge from the vertex with the lower time value $t_l$ to the vertex with the higher time value $t_h$, creating a tree-based network [47]. If the addition disturbed the tree-child property, we deleted the reticulation edge and contracted the vertices. Otherwise, we set the length of the reticulation edge to $t_h$ - $t_l$. The above procedure was repeated until *k* reticulations were successfully added.

Note that this way of introducing reticulation edges does not change time values of the leaves, hence all displayed trees of the resulting network are ultrametric and have equal heights.

*(ii) Simulating gene trees.* For each phylogenetic network, we randomly chose one of its base trees [47], obtaining one of the possible trees, along which gene families evolve. We simulated one gene tree per each base tree using SimPhy version 1.0.2 [48] with the following command: 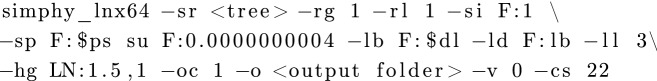
where<tree>is a nexus file containing the randomly chosen base tree, $ps is the effective population size and $dl is a duplication/loss rate. Similarly to [43], we used three rates of duplication/loss (DL) $$\{10^{-10}, 2\cdot 10^{-10}, 5\cdot 10^{-10}\}$$ and two values of the effective population size $$\{10^{7}, 5\cdot 10^{7}\}$$, corresponding respectively to a low and a medium level of incomplete lineage sorting (ILS). Altogether, we used six sets of simulation parameters, which allowed us to obtain a diversified set of gene trees. Note that low DL and ILS parameters were obtained based on biological data of *Saccharomycetales* fungi [44], whilst higher parameters were used to test the performance of the algorithms in a more challenging environment.

*(iii) Simulating sequences and estimating gene trees.* To introduce errors to the generated gene trees, we simulated sequences and estimated gene trees from multiple sequence alignments using the maximum-likelihood method (MLE). DNA sequences were simulated by INDELible v1.03 [49] by running a perl script INDELible_wrapper.pl included in SimPhy [48]. Again, we followed the parameters proposed in [43]. We used GTR model with substitution rates (AC, AG, AT, CG, CT and GT respectively) sampled for each gene tree from Dirichlet(12.776722, 20.869581, 5.647810, 9.863668, 30.679899, 3.199725). The nucleotide frequencies (T, C, A and G respectively) were sampled from Dirichlet (113.48869, 69.02545, 78.66144, 99.83793), whilst $\alpha$ parameter was sampled from Lognormal (−0.470703916, 0.348667224). The alignment length was set to 1000 bp. To estimate gene trees, we used a true alignment returned by INDELible. Then, we inferred ML-trees by PhyML v.3.1 [50] using GTR+$\Gamma$ model. Finally, to obtain a rooted gene tree from an unrooted ML-tree, we conducted midpoint-plateau rooting implemented in URec [51] using the corresponding displayed tree inferred in step (ii) of our pipeline.

Finally, for each set of parameters of duplication-loss rates, population sizes, reticulation values, and leaf-set sizes we simulated 100 networks and 100 corresponding gene trees.

**Fig. 5:**
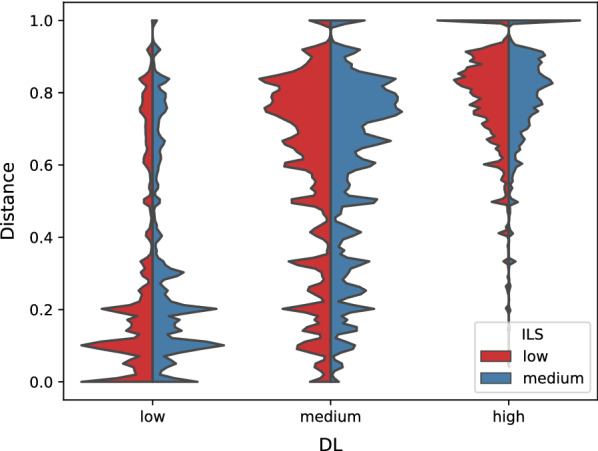
Distance between the simulated displayed trees and the corresponding gene trees for six sets of simulation parameters. For visualisation purposes we smoothed out the obtained histogram using a Gaussian kernel density estimate calculated by [59] with bandwidth set to 0.03.

**Fig. 6:**
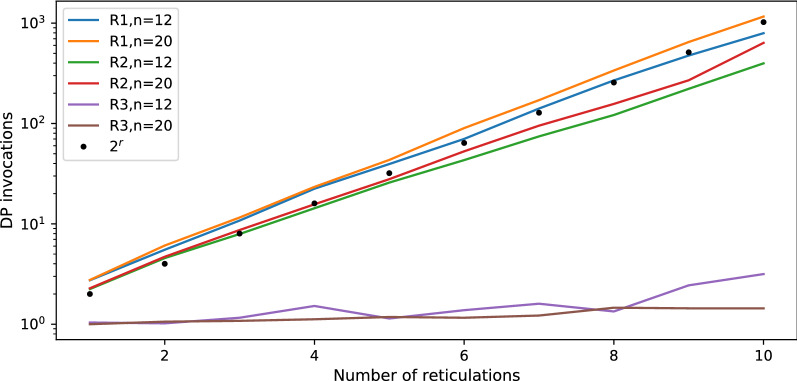
Average number of DP invocations necessary to calculate an answer for random datasets R1, R2 and R3.

Then, we examined the impact of duplication-loss rates and population sizes on the results of the simulation. We calculated a distance between the displayed tree used to simulate a gene tree in step (ii) of our simulation pipeline and the rooted ML gene tree for each sample. We used a strict metric implemented in [52], which was designed to compare species trees with gene trees in the presence of duplication events. The results are depicted in Fig. 5. We observe that the distances are significantly higher for high and medium DL rates than for the low DL rate. It suggests that increasing the parameter resulted in more demanding datasets for our algorithms than average empirical data. Interestingly, we see little difference between the dataset simulated using low and medium ILS parameters, suggesting that incomplete lineage sorting rarely changed the tree topology during our simulation study.

The simulations were run in parallel on ten cores and the total simulation time was under 8 h. The algorithm took 2 h to process all datasets, and it took, on average, 45 s to run 100 instances with 20 leaves and 10 reticulations for low ILS and low DL.

**Fig. 7:**
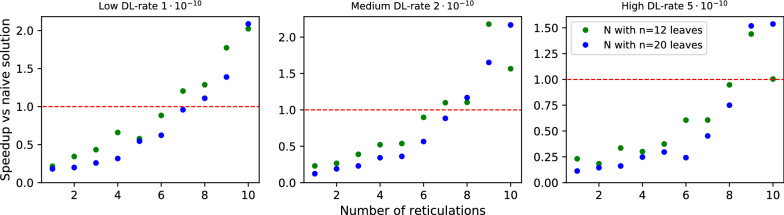
Performance of Algorithm 1 vs naïve approach for three simulated datasets with low ILS $1\cdot 10^{7}$.

**Fig. 8:**
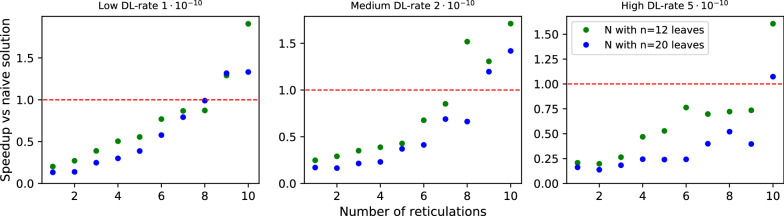
Performance of Algorithm 1 vs naïve approach for three simulated datasets with medium ILS $5\cdot 10^{7}$

#### Results for deep coalescence cost

*Discussion.* In Figs. 7 and 8, we present diagrams showing the results of evaluations for our datasets. The way we simulated data makes trees and networks more similar to each other. Thus, we can see significant improvements vs. random datasets. Regardless of parameter choices, we start to outperform the naïve solution for $r > 9$. For simulated data closest to reality (low ILS, low DL), we achieved better results for $r > 7$. The results suggest a hybrid approach in Algorithm 1: enumerate all displayed trees to compute $\mathsf {DC}$ costs directly if the network has a low number of reticulations (e.g., $r<9$).

**Fig. 9:**
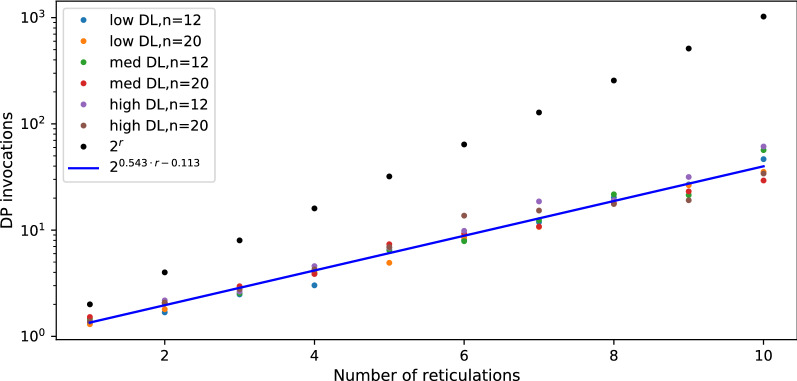
Average number of DP invocations necessary to calculate an answer for deep coalescence cost, for datasets with low ILS. The blue line represents coefficients calculated by linear regression for data with low and medium ILS combined.

**Fig. 10:**
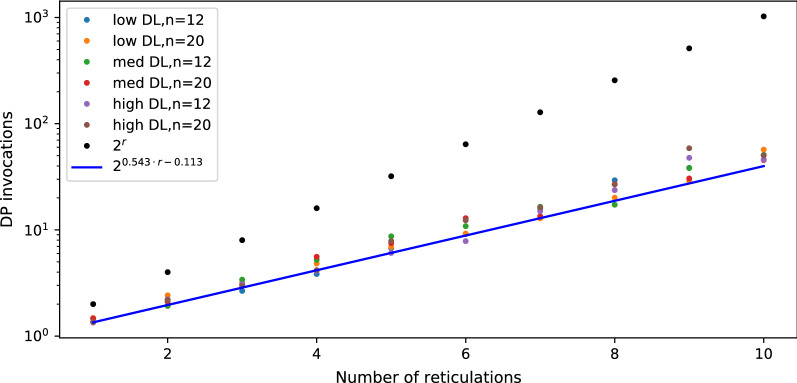
Average number of DP invocations necessary to calculate an answer for deep coalescence cost, for datasets with medium ILS. Recall that blue line represents coefficients calculated by linear regression for data with low and medium ILS combined.

To estimate the average runtime of our algorithm, we first calculated the depths *d* of the recursive calls as $\log _2$ of the number of DP invocations from each experiment. Then, we found that for all points $\langle r,d \rangle$ from our experiments, $d=0.543r-0.1135$ is the fitted least squares regression line having the standard error of .011 (see Figs. 9, 10). We conclude that, despite the worst case theoretical complexity, i.e., $O((2^{r+1}-1)|G||N|)$, the real runtime of our implementation on simulated data is proportional to $2^{0.543r}|G||N|$ and outperforms the naïve approach starting from small *r*’s. We claim that a similar statement holds for the algorithm with level-*k* networks. In other words, it is possible to analyse empirical networks even with $r=k=40$, since the exponent can be reduced by half.

#### Results for duplication cost

**Fig. 11:**
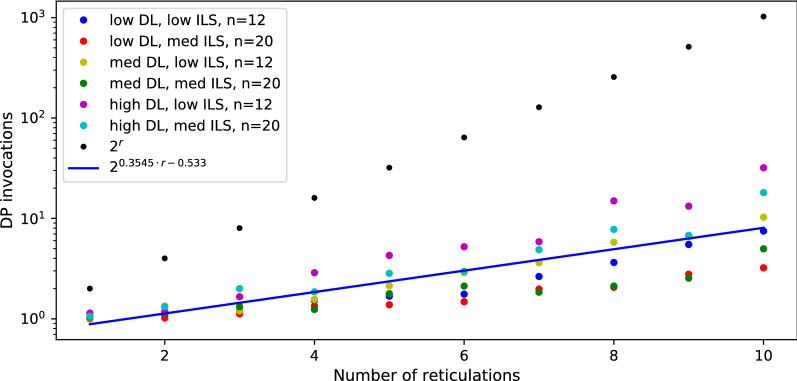
Average number of DP invocations necessary to calculate an answer for duplication cost, for datasets with low ILS. The blue line represents coefficients calculated by linear regression for data with low and medium ILS combined.

**Fig. 12:**
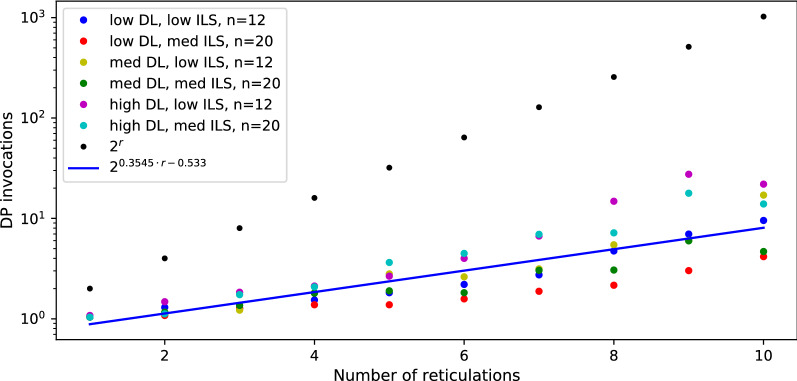
Average number of DP invocations necessary to calculate an answer for duplication cost, for datasets with medium ILS. Recall that blue line represents coefficients calculated by linear regression for data with low and medium ILS combined.

We also have conducted analogous experiments on the same datasets under the duplication cost and the implementation of dynamic programming formulas (15)-(21). In summary, our implementation outperforms naïve approach for $r > 5$. Using the same method to calculate the least squares regression line, we conclude that the average complexity for duplication is proportional to $2^{0.355r}|G||N|$ (see Figs. 11, 12; we omit the rest of the diagrams for brevity). Despite the same theoretical complexity of computing duplication and deep coalescence costs, dynamic programming formulas, and branch and bound strategies, we observe that the duplication cost has improved performance in practice in terms of the runtime and the exponent obtained in the regression formula. We claim that these improvements come directly from technically simpler formulas present in our algorithms under the duplication cost.

### Empirical tests

Our final experiment was conducted using real data. We revisited research presented in [53] concerning coronavirus (CoV) phylogeny. In the cited paper, the authors investigated the origins of the SARS-CoV-2 virus, which causes severe respiratory disease. They validated the hypothesis that the appearance of this new coronavirus is a consequence of several recombination events that occurred between some evolutionarily close CoV species. The results showed that both intergenic and intragenic recombination played a significant role in the SARS-CoV-2 evolution.

The goal of our study was to test whether scenarios with the lowest $\mathsf {DC}$ cost inferred for the phylogenetic network from [53] and individual gene trees confirm recombinations identified in the cited paper. In our experiment, we focused on intergenic recombinations, in which a whole gene is transferred from one species and integrated into another species genome.

*Data preparation:* In the gene trees inference step, we followed the cited work. We selected 15 out of 25 examined coronavirus species, omitting a few species to avoid multifurcations. As representatives of SARS-Cov-2, two variants were used. One was sampled from a patient from Wuhan (Hu-Wuhan), the origin of the pandemic spread of coronavirus, and the other was collected in Italy (Hu-Italy). Other selected species were RaTG13 bat CoV from *R. affinis* which, at first, was considered the only close relative of SARS-CoV-2, bat CoV ZC45 and ZXC21 strains from Zhejiang province of China (Bat-CoVZC45, and Bat-CoVZXC21), bat coronaviruses collected from species found in several provinces of China and from Bulgaria (Rf1, HKU3-12, BatCoV273, BatCoV279, and BM48-31 BGR), two CoV strains from Guangdong and Guangxi pangolins (Guangdong-Png, Guangxi-Png-P2V), and three SARS CoV related species (SARS, SARS-BJ182-4, and Rs3367). For the list of full names and database accession numbers, please refer to Table 1.

**Table 1:** List of the full names and database accession numbers of coronavirus species used in our research. Species were chosen from the dataset studied in [53]

Abbreviated name	Organism name	Accession Number (GenBank/GISAID)	Host organism
Hu-Wuhan	BetaCoV Wuhan-Hu-1	$NC_045512.2$	Human
Hu-Italy	hCoV-19/Italy/ ABR-IZSGC-TE4836/2020	$EPI_ISL_418260$	Human
RaTG13	Bat CoV RaTG13	MN996532.1	Bat
Guangdong-Png	hCoV-19/pangolin/ Guangdong/1/2019	$EPI_ISL_410721$	Pangolin
Guanxi-Png-P2V	Pangolin CoV isolate $PCoV_GX-P2V$	MT072864	Pangolin
Bat-CoVZC45	Bat SARS-like CoV isolate bat-SL-CoVZC45	MG772933.1	Bat
Bat-CoVZXC21	Bat SARS-like CoV isolate bat-SL-CoVZXC21	MG772934.1	Bat
Bat-CoV273	Bat CoV BtCoV/273/2005	DQ648856.1	Bat
Bat-CoV 279	Bat CoV BtCoV/279/2005	DQ648857.1	Bat
HKU3-12	Bat SARS CoV HKU3-12	GQ153547.1	Bat
Rf1	Bat SARS CoV Rf1	DQ412042.1	Bat
SARS	SARS CoV BJ01	AY278488.2	Human
SARS-BJ182-4	SARS CoV BJ182-4	EU371562	Human
Rs3367	Bat SARS-like CoV Rs3367	KC881006.1	Bat
BM48-31-BGR	Bat CoV BM48-31/ BGR/2008	GU190215.1	Bat

Coronavirus sequences were obtained from GenBank [54] and GISAID [55] databases. In the studied phylogenetic network, recombinations were found in the case of the genes M, ORF1ab, ORF3a, ORF6, ORF8, and ORF10; therefore, our research was focused on this set of genes. Multiple sequence alignments for the gene families were performed with MUSCLE [56] and corrected by GBlocks [57] with a less stringent correction option. The ML gene trees were inferred using RAxML [58] with parameters described in [53]. All species were present in all gene families except ORF8, which lacks the BM48-31-BGR species.

**Fig. 13:**
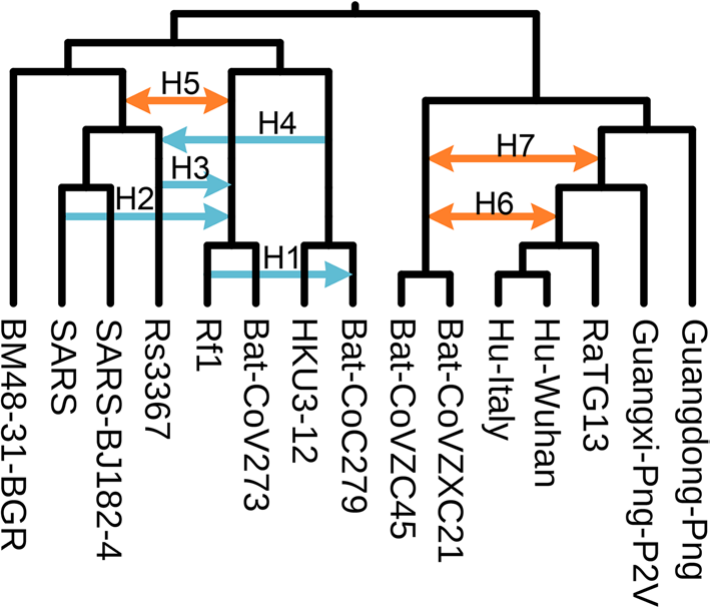
Coronavirus species tree with recombinations *H*1–*H*7 reported in [53]. Genes-recombinations assignment from [53]: *H*1:ORF1ab, *H*2:ORF8, *H*3:M, *H*4:ORF8, *H*5:ORF3a, *H*6:ORF6, *H*7:ORF3a, ORF8, ORF10.

*Phylogenetic networks:* The coronavirus tree with marked intergenic recombinations (*H*1-*H*7) identified in [53] is depicted in Fig. 13. Since the direction of three out of seven recombinations was not certain, we prepared 8 networks corresponding to all combinations of the directions of gene transfers. Each network is named with three letters *L*/*R* responding to the direction of *H*5, *H*6, and *H*7, respectively, i.e. in the *LRL* network, *H*5 and *H*7 are directed left, and *H*6 is directed right. Please note that the inferred networks are not tree-child, and therefore, in this experiment, we use the extended version of our algorithm described in Sect. *Beyond tree-child networks*.

**Fig. 14:**
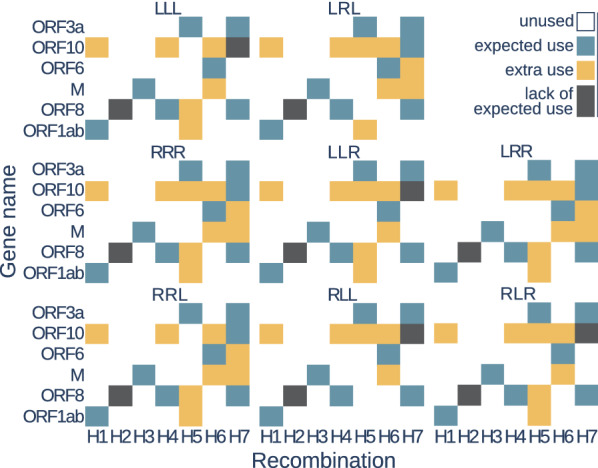
Results for coronavirus dataset showing which recombination edges were used by each gene tree in the scenarios with the lowest $\mathsf {DC}$ cost for each network variant.

*Discussion: *The results of the experiment are depicted in Fig. 14. For each gene family, we checked whether the expected reticulation edge was used by the inferred scenario with the lowest $\mathsf {DC}$ cost. We can distinguish three possibilities for reticulation edge *e*: 1. *e* was used by the expected gene, 2. *e* was used by one or more extra genes, and 3. the expected gene didn’t use *e*. We were able to confirm most of the reticulations except two: *H*2 that was reported in [53] with the lowest support wasn’t confirmed by any of the networks, and *H*7 was confirmed only by networks with *H*6 directed right. The most extra uses were found for *H*5 and gene *ORF*10, which gene tree had low support values. Further research might be performed for these cases. The least extra uses were present in *RLL*(5) and *LLL*(6), which may be some lead when investigating the direction of *H*6 and *H*7.

We observed that, for a fixed gene tree, the set of optimal displayed trees inferred by our algorithm and their cost, is independent of the network variant. This phenomenon needs further theoretical investigation. Costs for each gene tree have the following values: ORF3a: 0, ORF10: 5, ORF6: 1, M: 3, ORF8: 2, ORF1ab: 2. This observation may lead to discovering some important property and can be a subject for further investigation.

## Conclusions

In this work, we have investigated the problem of inferring an optimal tree displayed by a network under the deep coalescence and duplication costs. For each cost function, we proposed a new score function to approximate the cost. We have shown that these score functions have nice mathematical and computational properties allowing us to bound the cost of an optimal displayed tree from below. We have proposed a polynomial-time dynamic programming (DP) algorithm to compute the score together with the set of used reticulation edges that yielded the score. Then, we have proposed a new way to infer a displayed tree by a recursive procedure resolving conflicts detected in multiple invocations of DP. In the worst case, our algorithm to infer an optimal tree requires $2^{r+1}\text {-}1$ DP invocations, where *r* is the number of reticulations. However, numerous tests on simulated data have indicated that the exponent may be reduced by half on average. This phenomenon is explained by similarity, i.e., we expect a low number of conflicts if a gene tree is more congruent with its network. In other words, the average runtime of $\Omega (2^{0.543r}|G||N|)$ for the DC cost and $\Omega (2^{0.355r}|G||N|)$ for the duplication cost can compete on empirical datasets with exhaustive enumeration strategies (either on the level of a whole network or each biconnected component independently) commonly used in alternative approaches to scoring tree-network pairs [7, 9, 10]. We also claim that the statement holds for level-*k* networks by replacing *r* by *k* in the formula. We conclude that our conflict resolution algorithm enables analyses of complex networks with dozens of reticulation events. We also claim that resolving conflicts returned by dynamic programming is a new alternative towards designing efficient algorithms that utilize internal similarities of empirical datasets.

Resolving conflicts in the usage of reticulation edges can be naturally generalized to other cost functions, e.g., gene duplication and loss cost. Also, it is not difficult to extend DP to analyze unrooted gene trees. Another critical question is whether the runtime exponent can be further reduced, e.g., by choosing optimal scenarios with the smallest possible sets of conflicted reticulation edges. Also, the experimental results on the naïve approach justify the usage of a hybrid solution: when the recursion reaches networks with small number of reticulations, apply the brute force method instead of DP. Here, additional optimizations of the naïve approach could be applied; for instance, by improving displayed tree generator by adopting novel theoretical characterizations of stable networks [41].

Furthermore, we would like to test the efficiency and accuracy of the branch and bound algorithm to approximate the optimal cost. Also, we plan to apply the methods in computationally demanding problems of network inference from sets of gene trees, which may require reimplementation in a low-level programming language (e.g., C/C++).

## Data Availability

The software package and the simulated datasets are available at https://bitbucket.org/pgor17/embretnet.

## Abbreviations

DC: Deep coalescence
ODT: Optimal displayed tree
ODT-DC: Optimal displayed tree under deep coalescence
ODT-DUP: Optimal displayed tree under duplication cost
DP: Dynamic programming
DL: Duplication and loss

## Footnotes

1 Note that two leaves can be labeled by the same label in a binary network, which is not allowed in a phylogenetic network.

2 We also use $\sigma$ with edges, e.g., $\sigma (\langle v,w \rangle )$ denotes $\langle \sigma (v),\sigma (w) \rangle$.

3 Recall that N\X is the network obtained from *N* by removing all edges from *X*.
